# Chemical Pathology

**Published:** 1856-01

**Authors:** 


*Chemical Pathology—(From the Transactions of the Medical Associatiom of Southern Central New York.)*

Dr. J. G. Orton, of Binghampton, furnishes an excellent communication on Chemical Pathology. He shows the importance of an application of chemistry to the elucidation of the pathology of disease, with a view to the proper application of remedial agents. He particularly calls attention to the importance of chemical analysis of the renal secretion, quoting at the conclusion of his remarks on the subject, the following sentiment from Dr. Golding Bird:

"The analytical examination of the products of the renal secretion in disease, may be regarded as one of the most important aids in diagnosis, and which it would be alike injurious to the welfare of the patient, as to the credit and reputation of the practitioner, to avoid."

Dr. Orton concludes his essay with the following remark:

"The intimate connection which we have seen it evidently has with the science of medicine, and the important assistance which it will afford for the elucidation of the physiological and pathological phenomena manifested in the animal organism, and the rich harvest of honors which it will most assuredly yield to its assiduous cultivators, will ever commend it to the favorable consideration of every true lover of science as the 'guiding star' to the final separation of empiricism from medicine forever."

As a practical application of Dr. Orton's views to a case in practice, we quote the following from another portion of the *Transactions:*

*Oxalic Diathesis*, by J. G. Orton, M. D.—"Mr. W——, aged thirty-two, mechanic, consulted me June 16, 1854, giving the following history of himself during the past few years:

"Digestion uniformly disordered; obliged to take physic very frequently; appetite at times very deficient, but oftener exceedingly voracious, and peculiarly fond of saccharine substances; disquietude in sleep; easily frightened; much annoyed with diuresis; headache; palpitation of heart; depression of spirits, and general debility. An exacerbation of these symptoms takes place at least once every month, entirely unfitting him for physical or mental labor.

"At the time of the consultation, this gentleman was laboring under intense hypochondriasis, but free from pain or any local abnormal condition of the body that could possibly be detected by physical examination.

"*June* 17.—On examination of urina sanguinis, by chemical analysis and the aid of the microscope, I was enabled to record the following as its more important constituent elements: Specific gravity, 1.024; triple basic phosphates, excess; acid, medium; oxalate lime, very abundant in octohedral crystals; uric acid, deficient; urea, excess; mucus, excess. Pathology; oxalic diathesis.

"*Treatment.*—Hydrochloric acid, two drachms; nitric acid, one drachm; aqua pura, two ounces; ordered to be taken as follows: The acids to be separately mixed with half of the water, and twelve drops of each to be taken after having been mixed together, about half an hour before meals, three times a day. Ordered total abstinence from all saccharine substances, and in particular from pie-plant pies, and all articles of food calculated to increase the formation of oxalic acid in the system.

"*June* 20.—Patient reports himself as feeling much better; less headache; sleep more quiet; spirits buoyant, and gone to work at his trade. The urina sanguinis exhibited itself as follows, under examination: Specific gravity, 1.030; basic triple phosphates, medium; acid, medium: oxalate lime, less abundant; uric acid, deficient; mucus, less; urea, excess. Ordered a continuance of the acids.

"*June* 29.—Patient still on the improve; diuresis much abated, as all the other symptoms of functional disturbance.

"Analysis of urina sanguinis resulted as follows: Specific gravity, 1.028; basic triple phosphates, medium; acid, medium; oxalate of lime decreasing; uric acid, a trace; mucus, medium; urea, normal. A continuance in the use of the acids was ordered.

"*July* 19.—Patient reports himself as feeling extremely well; in every respect much better than he had been at any time for two years past. In fact, he complains of none of the symptoms which have been the cause of his uneasiness for years.

" On examination of the urina sanguinis, I found it as follows: Specific gravity, 1.028; basic triple phosphates, normal; acid, excess; oxalate of lime, absent; uric acid, medium; mucus, medium; urea, normal; quantity of excretion, normal. Ordered a discontinuance of the acids for the present, and prescribed vegetable tonics and zinc.

" Although I have discharged my patient for the present, it is probable that in the course of some months, a slight return of the *oxalic diathesis* will present itself, as is usual in this peculiar affection; in which case the acids will again be resorted to, and so on until, ultimately, the diathesis shall become permanently eradicated from the system.

" My patient was seen a few days since, and although nearly a year has now elapsed since he was first under treatment by the nitro-muriatic acid, yet he expresses himself as enjoying good health, and entirely free from all the troublesome symptoms he had formerly experienced.

" The point of interest in this case, is the same which is made manifest in all the cases of oxalic acid diathesis which have fallen under my observation, when treated by the nitro-muriatic acid: *provided* the crystals of oxalate of lime be of the *octohedral* form. If they be of the dumb-bell shape, the acid treatment will not effect a cure; the nitrate of silver then becomes the remedy of most value in its eradication.

" The microscope will alone decide what course of treatment it is proper to pursue."—*New Jersey Med. Reporter.*

